# Editorial: Immune cells and inflammatory mediators in mucosal pathologies, volume II

**DOI:** 10.3389/fimmu.2022.1092146

**Published:** 2022-12-06

**Authors:** Maria Leite-de-Moraes, Marcello Chieppa, Mónica Vermeulen

**Affiliations:** ^1^ Université Paris Cité, CNRS UMR 8253, INSERM U1151, Institute Necker Infant Malades, Paris, France; ^2^ Department of Biological and Environmental Sciences and Technologies (DISTEBA), University of Salento, Lecce, Italy; ^3^ Instituto de Medicina experimental (IMEX)-CONICET, Academia Nacional de Medicina, Buenos Aires, Argentina

**Keywords:** lung, inflammatory bowel disease, NOD receptors, IL-1β, epithelial cells

Mucosal tissues are the largest surface of exposed to the great heterogeneity of foreign antigens. This means that numerous mechanisms involving the innate immunity efficiently prevent the adherence and subsequent entry of most potentially pathogenic microorganisms. In this context, the epithelial cells play an essential role as the first mechanical and chemical barrier since, they play a selective role in the passage of macromolecules, ions, fluids and gas exchange at the level of the airways. Breakdown of this barrier leads to recognition of pathogen-associated molecular patterns (PAMPs) or damage signals (DAMPs) and subsequent activation of NFkB pathway inducing the secretion of cytokines initiating the inflammatory cascade.

Reviews and original research papers in this issue, provide novel approaches in relation to the mechanisms associated with the innate response both in lung and colonic tract. Given the various disorders affecting these tissues resulting in dysbiosis and a loss of normal homeostasis at the mucosa level, the contribution of new advances and novel approaches focused on the mechanism involved in early immune responses. These advances will be essential to allow better comprehension and management of diseases.

Several inflammatory mediators are implicated in mucosal pathologies. Among them, the nucleotide-binding domain (NOD)-like receptors (NLR) family plays an important role as intracellular sensors. In fact, NLRs sense microbial-associated and damage-associated molecular patterns and play a central role in maintaining tissue homeostasis and host defense against pathogens. The interesting review by Alvarez-Simon et al. is an update on the central role of NLRs in asthma, the most common airway disorder. They summarized the main features of NLRs, their importance in controlling immune responses in experimental models of asthma and their potential role in the pathogenesis of human disorder. The authors also described the therapeutic targeting of NLRs already tested in experimental models supporting the possible development of new therapies based on the modulation or inhibition of NLRs that could potentially be used as personalized medicine for certain patients.

In addition to asthmatic disorders, the lung mucosa is also a target for infections and the implication of NLRs were already reported. Here, Wang et al. demonstrate that *Pasteurella multocida*, one of the major pathogens inducing bovine respiratory syndromes causing high morbidity and mortality, induces inflammasome activation. This important defense mechanism against bacteria is activated in macrophages by the interaction of NLR3 belonging to the family of NOD receptors and the protein never in mitosis A(NIMA)-related kinase 7 (NEK7), which is a serine/threonine kinase. This interaction triggers the activation of the inflammasome and the subsequent production of IL-1β, in a potassium-dependent process. These data provided new insight on the *P. multocida*-induced host immune response.

Another example of airway infection is tuberculosis. In fact, the involvement of alveolar macrophages and innate or adaptive lymphocytes in airway inflammation observed following bacterial intracellular infection, including *Mycobacterium tuberculosis* (Mtb) is well documented in the literature. However, the contribution of neutrophils in Mtb infection is still controversial because in some conditions they can cause a protective and in others a pathological effect. Gaffney et al. review the neutrophil interactions with other immune cells, the metabolic pathways involved in neutrophil effector functions and discuss possible new therapeutic tools targeting host metabolism and function with a focus on Mtb infection.

The intestine is the largest mucosal organ of the body and also prone to various pathologies including colitis. Ulcerative colitis is a chronic intestinal inflammation that affects part of the colon and usually begins with inflammation of the rectum and spreads continuously. Its development is associated with disruption of the epithelial barrier and changes in the microbiota. IL-17-producing CD4+ T (Th17) cells are associated with the pathogenic inflammation. Here, Chang et al. demonstrate that the P2Y1 receptor, a G-coupled protein receptor, is abnormally increased in the splenocytes of mice with dextran sulfate-induced ulcerative colitis. This enhancement was associated with high expression of the transcriptional factor RORγt and IL-17A. Further, Th17 differentiation was impaired in P2Y1 deficient mice in an AMPK-dependent manner. This found is interesting because, during inflammation, macrophage, epithelial and endothelial cells release nucleotide phosphates (ATP and ADP) which accumulate as damage signals or DAMPs and interact with P2Y1 receptors to modulate immune responses namely activation of NLR3 inflammasome and T cells.

Another common pathology of the intestinal mucosa sometimes associated with colitis is food allergy. In this Issue, Vaccaro et al. assessed the mechanism that could contribute to eosinophil recruitment in colorectal tissues of food-sensitized pediatric patients. The authors found that eosinophils, CCL26, IL-4 and IL-13 were significantly up-regulated in polyps compared to control adjacent colorectal tissues. IL-13 was the main trigger of CCL26 production by epithelial cells through a STA3/STAT6/JAK1-2 pathway. These findings indicated that IL-13 produced by Th2 lymphocytes stimulates the release of CCL26 by epithelial cells thereby increasing the recruitment of eosinophils into the tissues. Overall, the data clearly suggest a pro-inflammatory role of epithelial cells in this disorder.

All of the authors in this issue contributed to a better understanding of the mechanisms deployed by innate cells at the mucosal level to deal with infectious agents or tissue damage. These interesting studies could allow the development of new therapeutic alternatives for the management of mucosal pathologies.

## Author contributions

ML-d-M, MC and MV edited the topic and wrote the manuscript. All authors contributed to the editorial and approved the submitted version.

